# ABO and Rhesus Blood Groups and Risk of Endometriosis in a French Caucasian Population of 633 Patients Living in the Same Geographic Area

**DOI:** 10.1155/2014/618964

**Published:** 2014-08-27

**Authors:** Bruno Borghese, Mélanie Chartier, Carlos Souza, Pietro Santulli, Marie-Christine Lafay-Pillet, Dominique de Ziegler, Charles Chapron

**Affiliations:** ^1^Université Paris Descartes, Sorbonne Paris Cité, Service de Gynécologie-Obstétrique 2 et Médecine de la Reproduction, Groupe Hospitalier Cochin, AP-HP, 75014 Paris, France; ^2^Institut Cochin, Université Paris Descartes, Sorbonne Paris Cité, CNRS (UMR 8104), 75014 Paris, France; ^3^Inserm, U1016, 75014 Paris, France; ^4^Serviço de Ginecologia e Obstetrícia (SGO), Hospital de Clinicas de Porto Alegre (HCPA), 90035-003 Porto Alegre, RS, Brazil

## Abstract

*Objectives*. The identification of epidemiological factors increasing the risk of endometriosis could shorten the time to diagnosis. Specific blood groups may be more common in patients with endometriosis. *Study Design*. We designed a cross-sectional study of 633 Caucasian women living in the same geographic area. Study group included 311 patients with histologically proven endometriosis. Control group included 322 patients without endometriosis as checked during surgery. Frequencies of ABO and Rhesus groups in the study and control groups were compared using univariate and multivariate analyses. *Results*. We observed a higher proportion of Rh-negative women in the study group, as compared to healthy controls. Multivariate analysis showed that Rh-negative women are twice as likely to develop endometriosis (aOR = 1.90; 95% CI: 1.20–2.90). There was no significant difference in ABO group distribution between patients and controls. There was no difference when taking into account either the clinical forms (superficial endometriosis, endometrioma, and deep infiltration endometriosis) or the rAFS stages. *Conclusion*. Rh-negative women are twice as likely to develop endometriosis. Chromosome 1p, which contains the genes coding for the Rhesus, could also harbor endometriosis susceptibility genes.

## 1. Introduction

Endometriosis is a chronic gynecological disease that severely affects quality of life [1]. High healthcare costs and repeated surgery are two hallmarks of the disease. One explanation for that is late diagnosis. The time between onset of symptoms and medical diagnosis is more than eight years in most industrialized countries [2]. Finding risk factors, especially for the most severe forms of the disease, is a crucial issue that may contribute to shortening the time from the initial symptoms to the diagnosis. Epidemiological risk factors for endometriosis have been consistently reported: a low body mass index (BMI), a family history of endometriosis, a personal history of severe and lasting dysmenorrhea at the time of adolescence, and the need to use oral contraceptives for alleviating dysmenorrhea that failed to respond to nonsteroidal anti-inflammatory drugs [3–5]. It is useful to gather this information when evaluating women experiencing infertility and/or pelvic pain [6].

With the exception of fetomaternal alloimmunization and hemolysins in relation to blood transfusion, the relationship between blood groups and human diseases, such as cancer or inflammatory diseases, has been controversial [7, 8]. To date three studies have investigated the association between endometriosis and blood groups, with varying results [9–11]. The discrepancies were so striking that an editorial has been published on this topic, reporting population stratification bias and ethnicity as potential explanations [12]. In addition, in these studies, the distinction between endometriosis subtypes has not been taken into account for the analyses. Yet, three forms of endometriosis are well recognized and are fundamentally different from each other: superficial peritoneal endometriosis (SUP), ovarian endometrioma (OMA), and deeply infiltrating endometriosis (DIE ) [13].

Consequently, we decided to set up a cross-sectional study with a design minimizing the abovementioned bias. We analyzed the blood groups distribution only in Caucasian women with and without endometriosis and originating from the same geographic area. We studied each clinical subtype (SUP, OMA, and DIE) separately. We also evaluated the frequency of ABO and Rhesus blood groups according to the revised American Fertility Society (rAFS) classification.

## 2. Materials and Methods

We conducted a cross-sectional study using data from a prospectively managed database. The structure of this database has already been detailed and published elsewhere [3]. Briefly we included all nonpregnant patients under 42 years old who were operated on (by laparotomy or operative laparoscopy) in our institution between January 2004 and November 2010. For the present study, we excluded from the cohort patients with cancer, non-Caucasian patients, and patients living outside Paris area (i.e., outside the region Île de France). Indications for surgery, sometimes more than one for each patient, were the following: (i) preoperative assessment of endometriosis by magnetic resonance imaging and/or ultrasound; (ii) pelvic pain, defined as the presence, for at least 6 months, of dysmenorrhea and/or intermenstrual pelvic pain and/or dyspareunia of moderate to severe intensity; (iii) infertility defined as at least 12 months of unprotected intercourse not resulting in pregnancy; (iv) pelvic mass (benign ovarian cyst, uterine myoma, etc.); and (v) others: uterine bleeding, request for tubal ligation, tubal infection, and so forth. Patients visually diagnosed with endometriosis but without histologic confirmation were considered to be ineligible to participate in the study. A total of 633 patients were diagnosed with the presence (*n* = 311, study group) or absence (*n* = 322, control group) of endometriosis. Depending on pathologic findings, endometriotic lesions were divided into SUP, OMA, and DIE, with the latter being defined as lesions that infiltrate the* muscularis propria* of bladder, vagina, intestine, or ureter [13]. Because these lesions are frequently associated [14], the final staging was given by the worst lesion found in each patient, that is, from least to most severe: SUP, OMA, and DIE. During surgery, extension of endometriosis was also scored according to the rAFS classification [15]. Control group included 322 patients without any lesion of endometriosis, as checked by an exhaustive inspection of the peritoneal surface and abdominopelvic organs at the time of surgery. Demographic data, medical and surgical history, type and duration of symptoms, and ethnicity were collected by face-to-face standardized questionnaires conducted by the surgeon during the month before surgery, as previously reported [16]. Information on ABO and Rh blood groups was obtained from medical records. If not available, blood groups were systematically determined by conventional techniques during the preoperative assessment.

We calculated the sample size by OpenEpi, Version 2, open source calculator-SSCohort (http://www.openepi.com/OE2.3/SampleSize/SSCohort.htm). Regarding the ABO groups, we assumed an odds ratio (OR) of 3.0 (data from [9]) between cases and controls, a type I error of 0.05, and a power of 0.80. We calculated the sample size to 320 (with a case-control ratio of 1 : 1). Regarding the Rhesus factor we assumed an OR of 0.6 (data from [10]) between cases and controls, a type I error of 0.05, and a power of 0.80. We calculated the sample size to 626 (ratio 1 : 1). Therefore, with 633 participants, our study appeared sufficiently dimensioned.

Statistical analysis was performed using SPSS 13.0 (SPSS, Chicago, IL). Continuous data were presented as mean ± standard deviation (SD). Student's* t*-tests were carried out when necessary. The chi-square and Fisher exact tests were used for categorical data. We used OR and corresponding 95% confidence intervals (CI) to compare the distribution of ABO groups and Rhesus factor in the entire study group (all endometriosis patients) and in the different forms and stages of endometriosis, as compared to the control group. We also performed an unconditional logistic regression to control for potential confounding factors, such as age, pain, BMI, blood groups, gravidity, and parity. We performed a stepwise logistic regression analysis, in which a *P* value of 0.5 was used as entry criteria and a *P* value of 0.2 was the threshold for the covariate to stay in the model [17]. A *P* value of <0.05 was considered to be statistically significant. Assuming a two-sided significance level of 95% and a power of 80%, this study is powered to detect a difference of 11% between the two groups with a total sample size of 633 and two equal groups. The institutional review board at our center approved the study protocol, and each individual signed an informed consent form. Standards for reporting of cross-sectional study have been followed in accordance with the STROBE statement (http://www.strobe-statement.org).

## 3. Results

Patients with histologically proven endometriosis (study group: *n* = 311) were distributed as follows: (i) SUP: 53 patients (17.0%); (ii) OMA: 110 patients (35.4%); (iii) DIE: 148 patients (47.6%). Classification according to the rAFS stages was as follows: (i) stage I: 56 patients (18.0%); (ii) stage II: 67 patients (21.5%); (iii) stage III: 100 patients (32.2%); (iv) stage IV: 88 patients (28.3%). Control group consisted of 322 patients without endometriosis at the time of surgery. Indications for surgery in the control group were as follows: (i) benign ovarian cysts (*n* = 99), (ii) uterine myomas (*n* = 98), (iii) chronic pelvic pain (*n* = 57), (iv) pelvic inflammatory disease (*n* = 5), (v) infertility (*n* = 47), (vi) ovarian torsion (*n* = 2), and (vii) others (*n* = 14). Significant differences between cases and controls were observed in gravidity, parity, weight, BMI, and preoperative pain scores, as expected [3, 5, 16]. Conversely, age, height, and infertility status were comparable in both groups.

The distribution of ABO and Rh blood groups in women with and without endometriosis is shown in Table 1. There was no significant difference in ABO groups between patients and controls. On the other hand, a statistically significant difference was detected for the Rhesus factor (*P* = 0.011, Pearson chi-square). Women with the disease were more frequently Rhesus negative group as compared to the controls (23.1% versus 15.2%, resp.). Crude OR for endometriosis among the Rhesus negative patients was 1.27 (95% CI: 1.07–1.52). After multivariate analysis, taking into account age, pain, BMI, ABO group, parity, and gravidity, the difference remained significant. Adjusted OR (aOR) for endometriosis was 1.90 (95% CI: 1.20–2.90). When combining Rhesus and ABO groups, no difference in distribution was observed between cases and controls (*P* = 0.138, Pearson chi-square) (Table 2).

The distribution of ABO and Rhesus blood groups among the different clinical forms of endometriosis (SUP, OMA, and DIE) is shown in Table 3. There was no difference between the groups, either for ABO group or Rhesus factor (*P* = 0.34 and 0.26, resp., Pearson chi-square).

Finally, analyses of blood groups distribution according to the rAFS classification did not reveal any statistically significant differences between the rAFS stages (Table 4).

## 4. Comment

In this cross-sectional study of 663 Caucasian patients living in Paris area and referred to our institution for surgery, we observed a higher proportion of Rh-negative women in patients with histologically proven endometriosis, as compared to healthy controls. After multivariate analysis, Rh-negative women are twice as likely to have endometriosis (aOR = 1.90; 95% CI: 1.20–2.90). This result is reported for the first time in a French Caucasian population. Data available for other populations are slightly different [9–11]. In a series of 231 patients with endometriosis and 166 women without endometriosis from the Yale University School of Medicine, Matalliotakis et al. found a 2.9-fold increased risk for endometriosis in women with blood group A (OR = 2.9; 95% CI: 1.85–4.52) [9]. In a Korean population of 186 women with endometriosis, Kim et al. found a preponderance of group A among women with endometriosis (OR = 1.6; 95% CI: 0.8–3.3). Demir et al. did not confirm this result in a Turkish population of 304 women with endometriosis and 42 controls [10, 11]. Regarding the Rhesus factor, only Demir et al. reported a higher proportion of Rh-positive women among women with endometriosis, as compared to healthy women (84 versus 76%, resp.; *P* = 0.03) [10]. As stressed by Tabei, these discrepancies are probably related to different frequencies of blood groups among subpopulations or ethnic groups [12].

When considering the severity of endometriosis, as defined by the rAFS classification, no study, including our study, has shown any statistically significant association between blood groups and disease stages. In the present study, we separated for the first time the clinical forms of endometriosis but could not find any association between blood groups and SUP, OMA, or DIE.

Some limitations in our study should be considered. First, the fact that all patients were submitted to surgery may introduce selection bias, as these women were probably the most severe cases. Unfortunately this point remains unsolved in most studies published on the subject [18]. It must be borne in mind that our results are only applicable to this specific population. Secondly, it is likely that our study was underpowered to detect a slight difference in subsets of patients, especially in SUP, where the number of patients was low. Further studies, targeting specific forms of the disease and including a large number of patients for each subtype, are needed to confirm our results. Lastly, all patients in the control group had a benign gynecological condition. Some of them could be associated to a specific blood group. However, the distribution of ABO and Rhesus groups among controls was similar to that of the French population, according to the French Blood Service data (http://www.dondusang.net/rewrite/article/3160/ about-blood/blood-group-basics/blood-groups.htm?idRubrique=1178). Also, in the study of Kim and colleagues, there was no significant association between blood groups and fibroids, ectopic pregnancy, or female infertility [11].

Beyond these issues, our study has specific strengths. With 633 patients recruited, our study has the largest sample size to date. The application of strict histological and surgical criteria allowed us a highly accurate and undoubted selection of cases and controls. Following the recommendations of Tabei et al., we selected the control group in the same population as the patient group, that is, in a population originating from the same geographic area and from the same ethnicity [12]. We included only Caucasian women living in Paris in this study because the frequency of blood groups may vary according to ethnicity and living area, as stressed by Tabei. Of course, this topic should be evaluated in other populations to have a global view of blood groups distribution in endometriosis. However, our results can probably be extended to the whole French population, since distribution of ABO and Rhesus groups in our control group was similar to that in France.

Obviously, it is in the nature of such an investigation, in which a link is sought but not prespecified as being biologically plausible, to find statistically significant associations quite by chance. The association that we found between Rh-negative factor and endometriosis was not strong enough (OR < 2) to suggest a causal relation. However, as the value of the aOR was not far from 2, it deserves at least consideration. In general, finding an excess of Rhesus negative subjects among patients with endometriosis suggests a genetic predisposition. Rhesus negativity has been reported as a potential risk factor for esophageal and gastric cancers [19, 20]. Rhesus negativity may also predispose to cancers in the lung [21], mouth [22], breast [23, 24], and endometrium [25]. As endometriosis shares some behavioral resemblance with tumor cells, including invasion, survival, evasion from immune clearance, or establishment of a blood supply, some common pathways may be implicated in patients with Rhesus negative phenotype [26]. Moreover, the Rh blood group locus is found on the short arm of chromosome 1 (1p34-36), at a site reported to constitute a susceptibility locus for skin malignant melanomas [27] and containing at least one tumor suppressor gene [28]. An increase in risk of malignant melanoma has been observed in Rh-negative subjects [29]. Incidentally, an association between endometriosis and cutaneous melanoma has been repeatedly observed [30–32]. Comparative genomic hybridization (CGH) analysis revealed loss of DNA copy number on 1p in SUP and OMA [33]. Finally, the potential effect of nitric oxide has been recently reported in Rh-negative subjects [19]. Nitric oxide has been implicated both in the development of endometriosis and in the neoplastic degeneration of OMA [34, 35].

In conclusion, Rhesus negativity could represent a risk factor for endometriosis in a Caucasian population. Biologic rationale for this association is consistent and could lead to the improvement of our knowledge of endometriosis pathogenesis. This observation could also contribute to shortening the time to diagnosis as being part of a more global score to predict the risk of endometriosis, which would include other known risk factors.

## Figures and Tables

**Table 1:** Distribution of ABO and Rh blood groups in women with and without endometriosis.

	Endometriosis *n* (%)	Controls *n* (%)	Crude OR (95% CI)	aOR (95% CI)^a^	*P* value^b^
ABO groups					
A	148 (47.5)	139 (43.2)	1.20 (0.86–1.68)	1.18 (0.82–1.70)	0.270
AB	10 (3.0)	10 (3.1)	1.10 (0.45–2.80)	1.24 (0.47–3.29)	0.780
B	32 (10.3)	36 (11.2)	1.01 (0.58–1.70)	1.04 (0.58–1.80)	0.980
O	121 (38.9)	137 (42.6)	1		
Rhesus					
Negative	72 (23.1%)	49 (15.2)	1.27 (1.07–1.52)	1.90 (1.20–2.90)	0.011
Positive	239 (76.9%)	273 (84.8)	1		
Total	**311**	**322**			

^a^Logistic binary regression (age, pain, BMI, blood type and rhesus, parity, and gestity); aOR: adjusted odds ratio.

^b^Pearson chi-square.

**Table 2:** Distribution of combined ABO and Rh blood groups in women with and without endometriosis.

	Endometriosis *n* (%)	Controls *n* (%)	Total *n* (%)	*P* value^a^
Blood group				
A positive	117 (37.6)	116 (36.0)	233 (36.8)	0.138
A negative	31 (10.0)	23 (7.1)	54 (8.5)	
B positive	22 (7.1)	32 (9.9)	54 (8.5)	
B negative	10 (3.2)	4 (1.2)	14 (2.2)	
AB positive	7 (2.3)	6 (1.9)	13 (2.1)	
AB negative	3 (1.0)	4 (1.2)	7 (1.1)	
O positive	93 (29.9)	119 (37.0)	212 (33.5)	
O negative	28 (9.0)	18 (5.6)	46 (7.3)	
Total	**311 (100.0)**	**322 (100.0)**	**633 (100.0)**	

^a^Pearson chi-square.

**Table 3:** Distribution of ABO and Rh blood groups in women with endometriosis according to the clinical subtypes of disease.

	SUP *n* (%)	OMA *n* (%)	DIE *n* (%)	*P* value^a^
ABO groups				0.34
A	28 (53)	47 (43)	73 (49)	
AB	2 (4)	6 (5)	2 (1)	
B	5 (9)	15 (14)	12 (8)	
O	18 (34)	42 (38)	61 (41)	
Rhesus				0.26
Positive	37 (70)	83 (75)	119 (80)	
Negative	16 (30)	27 (25)	29 (20)	
Total	**53**	**110**	**148**	

^a^Pearson chi-square.

SUP: superficial endometriosis; OMA: endometrioma; DIE: deep infiltrating endometriosis.

**Table 4:** Distribution of ABO and Rh blood groups in women with endometriosis according to the rAFS classification.

	rAFS I *n* (%)	rAFS II *n* (%)	rAFS III *n* (%)	rAFS IV *n* (%)	*P* value^a^
ABO groups					0.26
A	27 (48)	37 (55)	40 (40)	44 (51)	
AB	2 (5)	0 (0)	6 (6)	2 (2)	
B	4 (11)	6 (9)	16 (16)	7 (8)	
O	23 (36)	24 (36)	28 (38)	34 (39)	
Rhesus					0.79
Positive	41 (73)	51 (76)	80 (80)	66 (76)	
Negative	15 (27)	16 (24)	20 (20)	22 (24)	
Total	**66**	**67**	**100**	**88**	

^a^Pearson chi-square.

rAFS: revised American Fertility Society classification.
